# The effect of age on emotion processing in individuals with mood disorders and in healthy individuals

**DOI:** 10.3389/fpsyg.2024.1204204

**Published:** 2024-01-26

**Authors:** Vanessa Gray, William Moot, Christopher M. A. Frampton, Katie M. Douglas, Peter Gallagher, Jennifer Jordan, Janet D. Carter, Maree Inder, Marie Crowe, Virginia V. W. McIntosh, Richard J. Porter

**Affiliations:** ^1^Department of Psychological Medicine, University of Otago, Christchurch, New Zealand; ^2^Te Whatu Ora (Health New Zealand) Waitaha Canterbury, Christchurch, New Zealand; ^3^Translational and Clinical Research Institute, Faculty of Medical Sciences, Newcastle University, Newcastle upon Tyne, United Kingdom; ^4^School of Psychology Speech and Hearing, University of Canterbury, Christchurch, New Zealand

**Keywords:** emotion processing, aging, mood disorders, facial recognition, depression

## Abstract

**Introduction:**

Emotion processing is an essential part of interpersonal relationships and social interactions. Changes in emotion processing have been found in both mood disorders and in aging, however, the interaction between such factors has yet to be examined in detail. This is of interest due to the contrary nature of the changes observed in existing research - a negativity bias in mood disorders versus a positivity effect with aging. It is also unclear how changes in non-emotional cognitive function with aging and in mood disorders, interact with these biases.

**Methods and results:**

In individuals with mood disorders and in healthy control participants, we examined emotional processing and its relationship to age in detail. Data sets from two studies examining facial expression recognition were pooled. In one study, 98 currently depressed individuals (either unipolar or bipolar) were compared with 61 healthy control participants, and in the other, 100 people with bipolar disorder (in various mood states) were tested on the same facial expression recognition task. Repeated measures analysis of variance was used to examine the effects of age and mood disorder diagnosis alongside interactions between individual emotion, age, and mood disorder diagnosis. A positivity effect was associated with increasing age which was evident irrespective of the presence of mood disorder or current mood episode.

**Discussion:**

Results suggest a positivity effect occurring at a relatively early age but with no evidence of a bias toward negative emotions in mood disorder or specifically, in depressed episodes. The positivity effect in emotional processing in aging appears to occur even within people with mood disorders. Further research is needed to understand how this fits with negative biases seen in previous studies in mood disorders.

## Introduction

Emotion processing involves the ability to perceive, interpret, and generate responses to socially relevant stimuli, such as the intentions and behavior of others (Green et al., 2012). Being able to undertake these functions efficiently is an important part of interpersonal relationships and social interactions. The Cognitive Neuropsychological Hypothesis of Depression suggests that in depression, there is evidence of a bias toward negative emotional stimuli (Warren et al., 2015). This bias to attend to and recall more negative information and to have a more negative interpretation of social cues may then play a role in the maintenance of depression and susceptibility to relapse (Disner et al., 2011). However, this theory does not explain well episodes of mania in bipolar disorder (BD) or the tendency for people with BD to relapse into mood states of either pole. Neither is it easy to fit within this theory the increasing recognition of mixed mood states both in major depressive disorder (MDD) and BD (Stahl et al., 2017).

Studies do suggest that people with MDD perceive emotional stimuli as more negative and attend to and recall more negative information (Disner et al., 2011; Bourke et al., 2012; LeMoult and Gotlib, 2019). In BD, emotion processing findings are less consistent, partly due to methodological differences such as mood state at the time of testing. However, some research has shown that, compared with healthy participants, individuals with BD are both less accurate and slower at identifying facial expressions and this effect is most often seen for negative emotions (Vederman et al., 2012; Van Rheenen and Rossell, 2013; Van Rheenen et al., 2017; Miskowiak et al., 2019). These impairments have been seen in symptomatic and euthymic mood states, and therefore, may be trait-related (Miskowiak et al., 2019). However, although there seems to be a relationship with symptom severity, data regarding euthymia in MDD is limited (Krause et al., 2021). It has also been shown that several other variables have an effect on emotion processing in mood disorders (Krause et al., 2021). A limitation in the field has perhaps been that studies have tended to focus on small samples of participants with narrowly defined mood disorder diagnoses, while larger samples may be more suited to understanding the interplay of the mood disorder diagnosis and other factors, such as age.

In aging, a “positivity effect” in older adults has been found using multiple experimental paradigms (Carstensen and DeLiema, 2018). This has been demonstrated in memory for emotional material (Charles et al., 2003), autobiographical memory (Kennedy et al., 2004; Cuddy et al., 2017), working memory (Mikels et al., 2005), attention to emotional faces (Mather and Carstensen, 2003), and recall of facial expressions (Sava et al., 2017). While older adults have shown worse performance overall in identifying facial expressions, the ability to recognize positive facial expressions (most often *happiness*, but also *surprised* expressions) seems to be least affected, showing either smaller deficits, similarity, or better performance than their younger counterparts (Ruffman et al., 2008). However, it is not clear how these results intersect with the changes seen in mood disorders, as few studies have examined both age effects and mood disorders combined. In a recent systematic review (Gray et al., 2021), we examined studies of emotion processing in older people with mood disorders. However, data did not allow firm conclusions to be drawn.

While the positivity effect in older people is well-established, its origin is still debated. There are two main theories. First, the Socioemotional Selectivity Theory posits that as we age, our underlying motivations change, and consequently, how we process information changes (Carstensen, 2006). The theory suggests that as the time to the end of our life grows shorter, we focus more on positive aspects of life and ignore or attend less to negative information. Extrapolated to facial emotion recognition (FER), it could be theorized that more attention is given to positive expressions and less to negative ones, and as such, accuracy may be greater for positive expressions.

In contrast, the Dynamic Integration Theory considers the effect of reduced capacity for cognitive processing on emotional information processing (Labouvie-Vief, 2003). It suggests that negative affect requires higher cognitive demand to process (Pratto and John, 1991), so when cognitive capacity is reduced with aging, we attend less to negative stimuli as we do not have the reserve to process them. This process then results in the positivity effect seen in behavioral research (Labouvie-Vief et al., 2007). The Dynamic Integration Theory would then suggest that the positivity effect occurs most in those with greater cognitive impairment. This suggestion is not consistently endorsed by the current literature, leaving some questions as to the validity of this theory (Mather and Knight, 2005; Petrican et al., 2008). For example, in mood disorders, a percentage of patients have significantly reduced cognitive ability, depending on the exact clinical characteristics of the sample being investigated (Douglas et al., 2018). According to this theory, it would be hypothesized that this reduced cognitive ability would relate to difficulties processing negative emotional stimuli, thereby producing a positivity effect. This is, of course, the opposite of what is observed in younger people with mood disorders, while the situation in older people with mood disorders has not been well researched (Gray et al., 2021).

The FER paradigm has been used widely in research in mood disorders with data suggesting decreased accuracy in the identification of negative emotions and biases toward the perception of neutral expressions as negative in depressed participants when compared with healthy control participants using this task (Bourke et al., 2010). Additionally, generalized deficits in FER have been noted in both active mood states and euthymia in participants with BD, compared with healthy control participants (Samamé, 2013). The FER paradigm has also shown evidence of FER changes in aging (Ruffman et al., 2008).

Interaction between non-emotional cognition and emotion-based processing was included in this analysis due to the suggestion that non-emotional cognition is implicated in the process of emotion regulation and as such a disconnect between these could result in less effective emotion processing (Gotlib and Joormann, 2010; Joormann and Vanderlind, 2014). Examining the interactions between general and emotional cognition could provide insight into whether these processes are integrated or reliant on one another. Measures of cognitive processes shown to be consistently impaired in MDD, including verbal fluency, working memory, and psychomotor function, were included (Douglas et al., 2011). Cognitive deficits in MDD are also differentially influenced by aging (Hammar et al., 2022).

### The current study

We, therefore, aimed to combine data from two previous studies, which examined FER in people with mood disorders and matched healthy control participants, to examine the interaction between the negativity bias seen in mood disorders and the positivity effect found in aging, including the effects of mood disorder diagnosis, age, and non-emotional cognitive function (and any interactions between these factors).

### Hypotheses

The current study had two main hypotheses:

1. That with increasing age a positivity effect would be seen, whereby participants may be more accurate at responding to positive emotions and/or be less accurate at responding to negative emotional expressions.
2. A negativity bias would be found in participants with mood disorders on emotion processing compared with healthy control participants.

Further to this we planned to conduct an exploratory analysis of the correlation between aspects of non-emotional cognition and FER with the hypothesis that there would be some correlation between aspects of non-emotional cognition and FER.

## Methods

### Data sets

Baseline data (single time-point) from two studies conducted at the Department of Psychological Medicine, University of Otago, Christchurch were used (Bourke et al., 2012; Carter et al., 2013; Inder et al., 2015).

#### Study 1

The first data set (Study 1) consisted of 98 people who were experiencing a Major Depressive Episode (MDE) either in the context of BD (*n* = 8; BD II only) or MDD (*n* = 90) and healthy control participants (*n* = 61), aged 18 to 65 years. Mean age was 38.2 years (mood disorder = 38.5 years, healthy control = 37.7 years; Bourke et al., 2012; Carter et al., 2013). Ethics approval was granted by the Upper South Canterbury Ethics Committee of NZ and this trial was registered with the Australian and New Zealand Clinical Trials Registry (ACTRN12605000723684). No further ethics approval was required for the current analysis as the use of deidentified data for future analysis was consented to a recruitment.

The Structured Clinical Interview for DSM–IV Axis I Disorders–Research Version (SCID–I–RV) was used to confirm a mood disorder diagnosis (First et al., 1998). Exclusion criteria were schizophrenia, BD I, current serious alcohol or drug misuse or dependence, neurological illness (e.g., epilepsy), and pregnancy. Participants needed to be free of any centrally active drug, other than the occasional hypnotic, and the oral contraceptive pill, for a minimum of 6 weeks. Participants were referred by general practitioners and mental health services or could self-refer. The Montgomery-Asberg Depression Rating Scale (MADRS) was used to measure depressive symptoms in both studies (Montgomery and Åsberg, 1979). The scores of depressed participants in Study One were in the moderate range of depression (Mean = 24.2).

The healthy control group consisted of age and gender-matched psychologically healthy individuals without a personal history, or a history in a first-degree relative, of major mental illness.

#### Study 2

The second data set (Study 2) consisted of 100 people with a diagnosis of BD (I or II) aged between 16 and 36 years, with a mean age of 26.5 years (Inder et al., 2015). Ethics approval was granted by the Canterbury Ethics Committee of NZ and this trial was registered with the Australian and New Zealand Clinical Trials Registry (ACTRN12605000722695). No further ethics approval was required for the current analysis as the use of deidentified data for future analysis was consented to a recruitment.

As in Study 1, the SCID–I–RV was used to confirm a mood disorder diagnosis. Participants could be in any mood state at entry. Participants were recruited from a range of services, including mental health services and general practitioners. Exclusion criteria were minimal, including only alcohol or drug dependence as a principal diagnosis (Inder et al., 2015). The MADRS scores of BD participants in Study Two were in the mild range of depression [Mean = 15.7 (IPSRT group) and 13.3 (SSC group)].

#### Analysis groups

For the first analysis, healthy control subjects were compared with patients with mood disorders from both studies. This therefore included 198 participants with either MDD or BD. For the second analysis, the comparison was with participants who had current MDE–either in the context of MDD or BD (135 participants).

### Cognitive measures

The following tasks were administered in both studies and were included in the analysis.

#### Facial emotion recognition task

A computerized FER Task developed by Harmer and colleagues at Oxford University was used to examine emotion recognition (Harmer et al., 2003). A task using the Pictures of Affect Series was chosen over various other task options as this task is in accord with what has been used by others in the field [e.g., Harmer et al. (2009)] and as such would be able to produce further data applicable to exploration of the Cognitive Neuropsychological Hypothesis.

This FER task uses the six basic facial emotions, as described by Ekman and Friesen (1976); *anger*, *disgust*, *happiness*, *sadness*, *surprise*, and *fear*, as well as neutral expressions. As is convention and as represented in work on the Cognitive Neuropsychological Hypothesis, *happiness* and *surprise* are considered to be positive emotional expressions, and *anger, fear, sadness,* and *disgust*, negative expressions (Harmer et al., 2009).

The pictures presented to participants come from the Pictures of Affect Series (Ekman and Friesen, 1976) with each picture morphed between the original (full emotion) and neutral expression at 10% intervals, using methods described by Young et al. (1997). The pictures are black and white. Four examples of each of the six emotion categories (portrayed by male and female actors) at each level of intensity (10–100%) are presented (40 stimuli for each emotion). Each face is also presented in a neutral expression (24 stimuli), giving a total of 264 stimulus presentations.

The task is presented in three blocks (88 stimulus presentations per block) with an untimed rest period between each block to prevent fatigue. Each face is presented in random order for 500 ms and is then immediately replaced with a blank screen. On identification of the emotional expression, participants need to press the corresponding labeled key on a response pad. Participants were instructed to respond to each facial expression as quickly and accurately as possible. Accuracy and mean reaction time (ms) data were collected.

### Non-emotional cognitive tasks

#### Cambridge neuropsychological test automated battery tasks

The Cambridge Neuropsychological Test Automated Battery (CANTAB®) was used for many of the cognitive tasks (Sahakian and Owen, 1992). The CANTAB is a battery of non-verbal computerized cognitive tests administered with the aid of a touch-sensitive screen for reaction timing. Selected from the suite of tests were the following:

##### Motor screening

The motor screening test records the mean reaction time to respond to 10 pink or green crosses on the screen. This task is used both as training for the CANTAB tests and to exclude motor or visual disorders. The outcome recorded is the speed of response.

##### Spatial recognition memory

This task assesses the ability to remember the spatial location of visual stimuli (squares). Five squares are presented sequentially at different locations on the screen, then participants are presented with a pair of squares in counterbalanced order. They are instructed to identify which square is at a location where one was previously presented. The outcomes recorded are accuracy and speed.

##### Spatial working memory

Participants are asked to search through boxes on the screen to find which one hides a colored token. In doing this, they are required to remember where the tokens were previously placed. This task begins with four trials of four boxes and progresses to four trials of six, then eight boxes. Repetitious search errors were reported and a performance index for search strategy was generated.

##### Spatial span

Participants are required to remember, then replicate, the order of nine white squares on-screen that change color one by one. Trials progress from two to nine squares and the task self-terminates after three successive trial failures (incorrect sequence) on a given number of squares. The longest span length correctly recalled was reported.

#### Pen-and–paper tasks

##### The national adult reading test

This task was used as an estimate of premorbid IQ (Nelson and Willison, 1991). The National Adult Reading Test (NART) requires participants to pronounce a list of 50 English words, printed in order of increasing difficulty.

##### Rey auditory-verbal learning test

This task involves repeated learning and recall trials of a 15-word list (Rey, 1964). In the described studies, words are pre-recorded and presented over computer speakers for consistency. Task administration involves the auditory presentation of a list of 15 non-related words over five acquisition trials, followed by recall after each trial. A second distracter list of 15 different non-related words is then presented, and immediately after recall of this list, a sixth recall trial of the first list follows. After 20 min (during which other, non-verbal tasks are completed), delayed recall of the first list is tested. Following the delayed recall of the first list, the recognition trial is completed. The recognition component of the Rey Auditory-Verbal Learning Test (RAVLT) is presented as a computerized task. Primary outcomes were total words recalled in the first five trials and total correct words recalled in a delayed recognition trial.

##### Controlled oral word association test

Participants are required to generate as many words as possible beginning with a particular letter over a one-minute period (Benton, 1983). Three trials are completed using letters F, A, and S. Proper nouns, place names, or the same word with a different ending are excluded. The primary outcome was the total number of correct words generated across the three trials.

##### Digit span forwards and backwards

This task requires participants to repeat increasingly longer strings of numbers either forward, as presented, or backward, in the reverse order, after they are read aloud by the examiner (Wechsler, 1997). The primary outcome was the longest correct number of digits recalled by the participant in each condition.

### Statistical analysis

Statistical analyses were conducted using the IBM SPSS Statistics (Version 28). Demographic and clinical data were summarized using standard descriptive statistics. Comparison of demographic and clinical variables between healthy control participants, MDD patients, and BD patients used chi-square tests for categorial measures, and ANOVA for continuous measures, with Fisher’s Protected Least Significant Difference and post-hoc chi-square tests for pairwise comparisons.

Three variables related to FER were examined: (a) accuracy of emotion recognition, (b) reaction time, and (c) a Performance Index (PI). The PI was calculated as follows. FER accuracy and reaction time items were Z-transformed. Z-scores outside a range of ±2.5SD were excluded from further analysis as these outlying scores likely represent an impairment with that task which cannot be accounted for due to normal variation, such as a misunderstanding of instruction or individual cognitive impairment. This exclusion range was chosen after examining the data distribution and making a pragmatic decision based on the number of outliers. The Z-score for reaction time was then subtracted from that for accuracy with a higher score reflecting a combination of more rapid and accurate performance and, conversely, a lower score, slower and more incorrect responses. The formula used for this calculation is expressed below:

PI=Zac−Zrt

Using this formula, a participant who is performing very accurately and quickly might have a score of +2 (or above), and a patient performing very accurately and slowly might have a score of −2 (or below).

In examining the cognitive data, to rationalize the effects of co-linear variables, we completed a Principal Components Analysis (PCA) using Varimax rotation. Components that explained >5% variability in the data were considered to be valid components of the data set. Standardized Component Scores were then used in the analysis. Variables that did not load on these components were analyzed separately. Three factors emerged–a verbal memory factor incorporating RAVLT total words remembered from lists 1–5 and RAVLT delayed recall (termed Verbal Memory Component); a factor involving spatial working memory and incorporating CANTAB spatial working memory between errors and spatial span (termed Spatial Working Memory Component); and a digit span factor incorporating digit span forward and digit span backward (termed Verbal Working Memory Component). Verbal fluency and motor screening latency did not load on these factors and were thus examined and presented as separate variables.

Repeated measures ANOVA was used to examine the effects of emotion, age, and group (control vs. mood disorder) and ‘emotion by age’ and ‘emotion by group’ interactions. Initially, NART was included in the analysis but was not significant either as a factor or in interactions. It was therefore dropped from the analysis. Where there was a significant interaction, ANOVA was used to further examine the effects of age and group on separate emotions. Effect sizes were calculated using Cohen’s *d*.

To examine the effect of participants being currently depressed, analyses were repeated as above however, including only those patients who were currently in an MDE, either unipolar or bipolar, as one group (MDE), and healthy control participants as the other group.

Finally, to examine the moderating effect of non-emotional cognitive function, partial correlations were calculated between FER variables and cognitive variables, controlling for age. The cognitive variables used were the factors from the PCA (Verbal Memory, Spatial Working Memory, Verbal Working Memory) and the variables that did not load on the PCA (verbal fluency, motor screening latency).

## Results

Descriptive statistics are shown in Table 1. There was a significant difference in age between the groups, with the BD group being significantly younger than the MDD and healthy control groups. This was not unexpected and is attributable to differences in the samples recruited in each original study.

**Table 1:** Demographic and clinical characteristics.

	Healthy Controls (*N* = 61)		Major Depressive Disorder (*N* = 90)		Bipolar Disorder (*N* = 108)	
	*N* (%)	*M* (SD)	*N* (%)	*M* (SD)	*N* (%)	*M* (SD)
Age	–	37.7 (12.7)^*^	–	38.6 (11.3)^*^	–	27.3 (7.0)^*^
MADRS	–	–	–	23.8 (6.7)	–	15.3 (10.7)
Gender (F)	41 (67)	–	61 (68)	–	79 (75)	–
Ethnicity (Pākehā)	50 (82)	–	70 (78)	–	90 (83)	–
Premorbid IQ (NART)	–	108.6 (7.65)*	–	107.1 (8.9)*	–	104.5 (8.4)*
Years of tertiary education	–	2.74 (1.9)*	–	2.71 (1.89)*	–	1.9 (1.94)*
Medication						
Lithium	–	–	–	–	29 (27)	–
Other mood stabilizer	–	–	–	–	41 (38)	–
Antipsychotic	–	–	–	–	48 (44)	–
Antidepressant	–	–	–	–	55 (51)	–
Bipolar I diagnosis	–	–	–	–	81 (75)	–

*Reflects significantly different at the 0.05 level. Eight participants in Study 1 had a diagnosis of bipolar depression and are therefore included in the BD group. F, Female; MADRS, Montgomery-Asberg Depression Rating Scale; NART, National Adult Reading Test.

### Associations between age, mood disorder diagnosis, and facial emotion recognition variables

Repeated measures ANOVA showed a significant interaction between age and emotion for accuracy (df 10,1,275; *F* = 8.1, *p* < 0.001), reaction time (df 10, 1,275; *F* = 5.9; *p* < 0.001), and PI (df 10, 1,275; *F* = 9.8; *p* < 0.001). There was no group by emotion interaction and no main effect of group (mood disorder vs. healthy control) for accuracy, reaction time, or PI.

**Figure 1:**
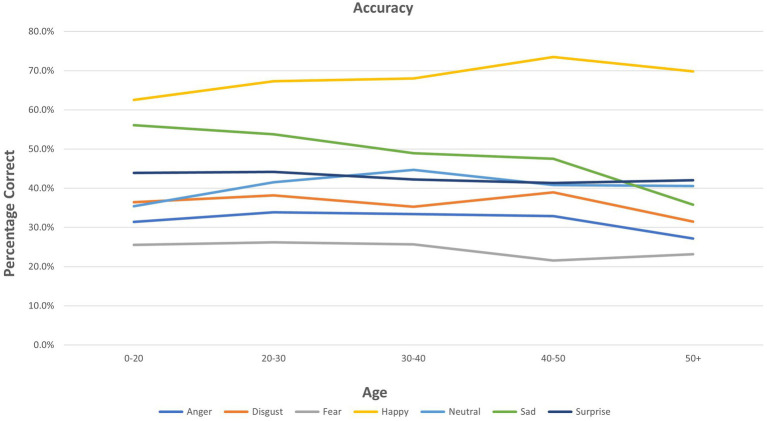
Accuracy of facial emotion recognition for each emotion by age.

**Figure 2:**
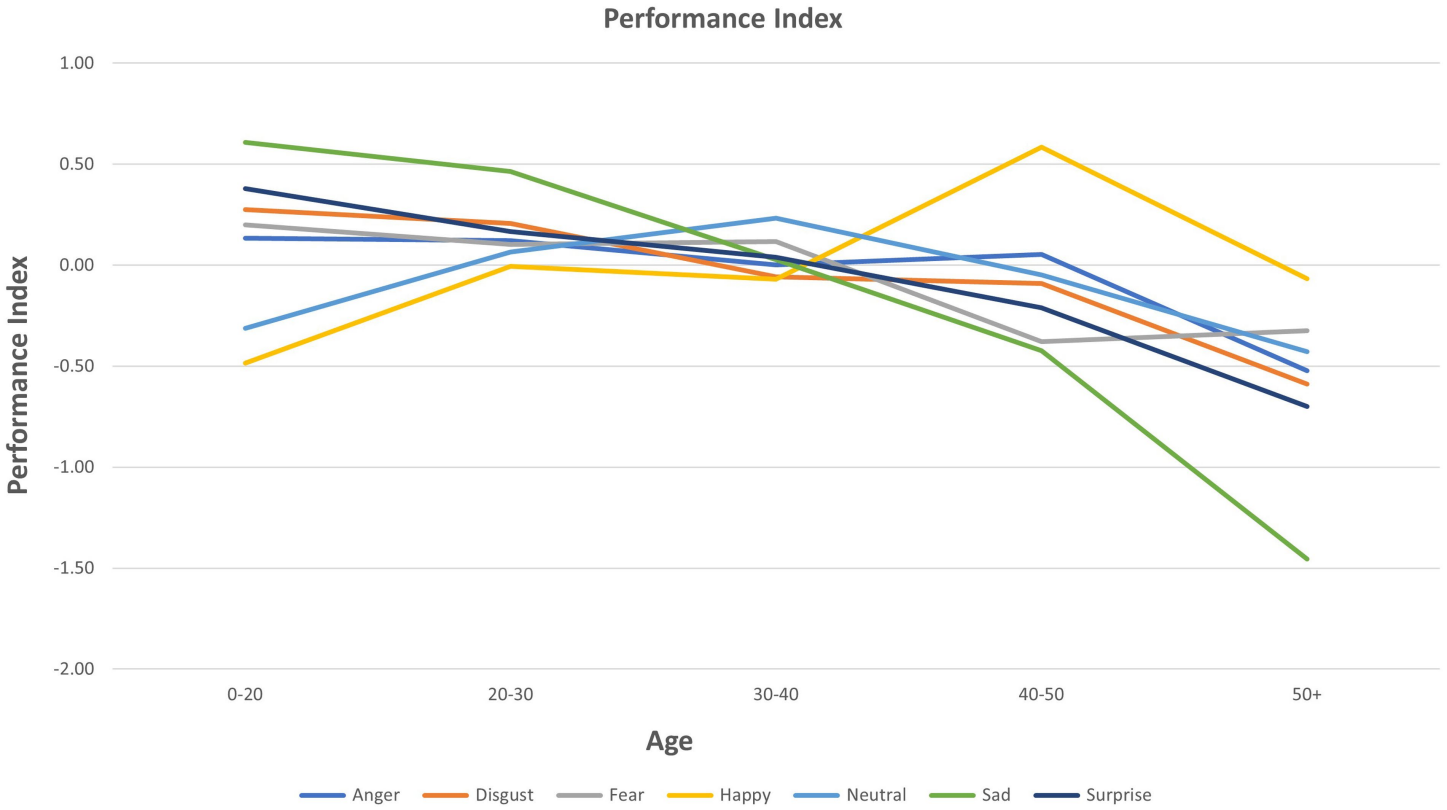
Reaction time for facial emotion recognition of each emotion by age.

ANOVA results for individual emotions are presented in Table 2. Age showed multiple significant relationships for the three FER outcomes (see also Figure 1). A significant group (mood disorder vs. healthy control) by age interaction was found both for accuracy and PI for *anger* (see Supplementary Figure S1). Adding the interaction ‘group by emotion’ did not affect the results for any other emotions (Figure 2).

**Table 2:** Effects of age and mood disorder on facial emotion recognition.

	Accuracy					Reaction Time				Performance Index		
	Group	Age			Group	Age			Group	Age		
	*F (p)*	*d*	*F (p)*	*B*	*F (p)*	*d*	*F (p)*	*B*	*F (p)*	*d*	*F (p)*	*B*
Anger	1.69 (0.19)	−0.004	**5.62 (0.02)**	**−0.002**	0.35 (0.70)	−0.05	**4.11 (0.04)**	**8.6**	1.52 (0.22)	0.03	**8.68 (0.004)**	**−0.03**
Disgust	0.34 (0.71)	0.12	2.12 (0.15)	−0.001	1.16 (0.32)	0.02	**10.50 (0.001)**	**12**	0.50 (0.61)	0.07	**10.79 (0.001)**	**−0.03**
Fear	0.99 (0.38)	0.13	**4.42 (0.04)**	**−0.002**	1.10 (0.34)	−0.04	**4.74 (0.03)**	**7.9**	2.25 (0.11)	0.13	**10.57 (0.001)**	**−0.03**
Happiness	0.21 (0.81)	0.09	**8.17 (0.005)**	**0.002**	1.12 (0.33)	−0.02	2.36 (0.13)	4.3	0.66 (0.52)	0.07	0.75 (0.39)	0.008
Sadness	0.72 (0.49)	−0.11	**26.9 (<0.001)**	**−0.005**	1.38 (0.26)	0.02	**26.61 (<0.001)**	**17.4**	1.76 (0.18)	−0.09	**48.77 (<0.001)**	**−0.06**
Surprise	0.31 (0.73)	0.06	1.90 (0.17)	−0.001	0.59 (0.56)	−0.03	**14.40 (<0.001)**	**11.2**	1.01 (0.37)	0.07	**15.31 (<0.001)**	**−0.03**

Significant outcomes in bold; Group: Control *n* = 61, Mood Disorder *n* = 198; *d* = Effect size of between group differences.

**Figure 3:**
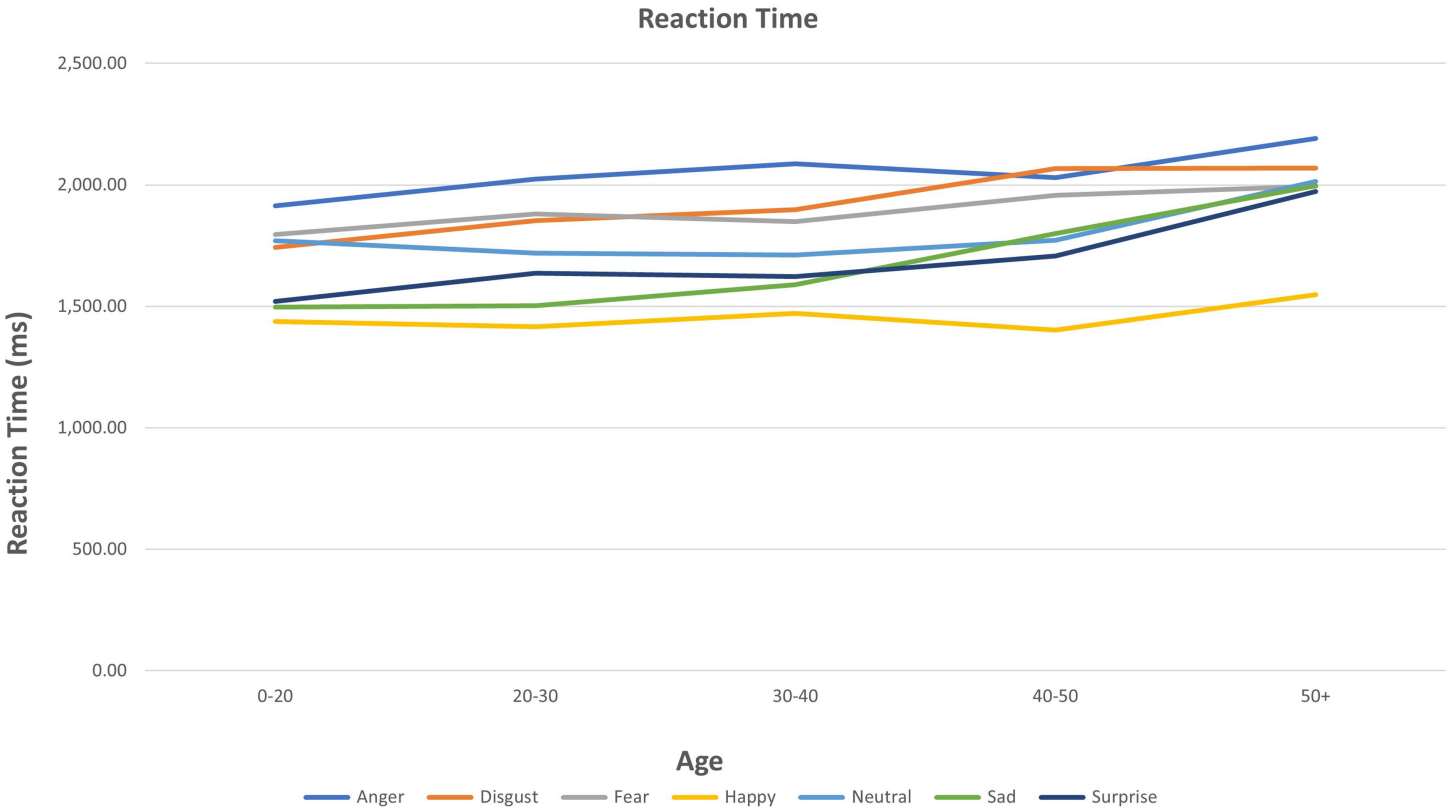
Performance index of facial emotion recognition for each emotion by age.

Scatter plots of Age, Group, and FER variables for each emotion are presented in Supplementary Figure S2 for interactions that were significant as per Table 2. These plots are provided to give a visual representation of the interactions referred to above (Figure 3).

### Relationship between age, current depression, and facial emotion recognition variables

Repeated measures ANOVA with current depression (MDE) as a factor (healthy controls, *n* = 61, MDE, *n* = 135) showed a significant interaction between age and emotion for accuracy (df 5,965; *F* = 8.6, *p* < 0.001), reaction time (df 5, 965; *F* = 6.5; *p* < 0.001) and PI (df 5, 965; *F* = 9.7; *p* < 0.001). There was no group by emotion interaction and no individual effect of group for accuracy, response time, or PI.

ANOVA for individual emotions is presented in Table 3. Age showed multiple significant relationships for the FER three outcomes. Adding the interaction ‘group by emotion’ did not affect the results for any emotions.

**Table 3:** Effects of age and current depression on facial emotion recognition.

	Accuracy				Reaction Time				Performance Index			
	Group	Age			Group	Age			Group	Age		
	*F (p)*	*d*	*F (p)*	*B*	*F (p)*	*d*	*F (p)*	*B*	*F (p)*	*d*	*F (p)*	*B*
Anger	0.260 (0.61)	−0.08	**5.30 (0.02)**	**−0.002**	0.01 (0.94)	−0.01	**4.23 (0.04)**	**8.52**	0.09 (0.77)	−0.05	**8.57 (0.004)**	**−0.027**
Disgust	1.24 (0.27)	0.17	1.17 (0.28)	−0.001	0.10 (0.75)	0.05	**8.16 (0.005)**	**10.32**	0.36 (0.55)	0.09	**7.60 (0.006)**	**−0.024**
Fear	1.17 (0.28)	0.17	**4.35 (0.04)**	**−0.002**	0.001 (0.97)	−0.01	2.65 (0.11)	5.95	0.63 (0.43)	0.12	**7.42 (0.007)**	**−0.022**
Happiness	0.12 (0.73)	0.05	**6.64 (0.01)**	**0.002**	0.05 (0.83)	0.03	2.02 (0.16)	4.12	0.005 (0.94)	0.01	0.47 (0.50)	0.007
Sadness	0.90 (0.35)	−0.15	**32.42 (<0.001)**	**−0.005**	0.21 (0.65)	0.07	**28.21 (<0.001)**	**17.75**	0.90 (0.35)	−0.15	**55.69 (<0.001)**	**−0.06**
Surprise	0.24 (0.63)	0.08	0.79 (0.38)	−0.001	0.01 (0.92)	−0.02	**13.49 (<0.001)**	**11.02**	0.20 (0.66)	0.07	**11.59 (0.001)**	**−0.03**

Significant outcomes in bold; Group: Control *n* = 61, Depressed Mood *n* = 135; d = Effect size of between group differences.

### Relationship between facial emotion recognition variables and non-emotional cognitive variables

Table 4 presents the result of partial correlations (adjusted for age) between FER accuracy, reaction time, and PI, and non-emotional cognitive variables (three PCA Components and the individual cognitive variables of verbal fluency and motor screening). The influence of group was not examined given the lack of effect of group in the analysis of FER.

**Table 4:** Partial correlations of non-emotional cognitive variables and age for accuracy, reaction time, and performance index.

	Verbal Fluency	Motor Screening	Verbal Memory Component	Spatial Working Memory Component	Verbal Working Memory Component
Accuracy r(*p*)					
Anger	0.03 (0.68)	−0.05 (0.40)	0.04 (0.49)	−0.11 (0.08)	−0.05 (0.40)
Disgust	**0.20 (0.002)**	−0.05 (0.45)	0.05 (0.48)	−0.12 (0.05)	**0.14 (0.03)**
Fear	0.11 (0.08)	0.05 (0.47)	0.06 (0.34)	−0.12 (0.07)	0.10 (0.11)
Happiness	0.06 (0.35)	0.01 (0.88)	0.01 (0.84)	0.02 (0.80)	0.02 (0.71)
Sadness	0.10 (0.58)	−0.10 (0.10)	**0.13 (0.05)**	−0.04 (0.53)	0.03 (0.65)
Surprise	0.03 (0.62)	−0.01 (0.91)	**0.13 (0.03)**	−0.04 (0.52)	0.09 (0.17)
Reaction Time r(*p*)					
Anger	−0.07 (0.24)	0.09 (0.16)	0.01 (0.93)	0.05 (0.43)	**0.13 (0.04)**
Disgust	**−0.14 (0.02)**	0.04 (0.48)	−0.02 (0.75)	0.04 (0.54)	0.07 (0.27)
Fear	−0.10 (0.18)	0.04 (0.51)	−0.03 (0.62)	0.04 (0.53)	0.09 (0.18)
Happiness	**−0.22 (0.00)**	0.02 (0.09)	−0.04 (0.50)	0.12 (0.06)	0.01 (0.90)
Sadness	−0.08 (0.19)	0.09 (0.18)	−0.03 (0.60)	**0.14 (0.03)**	0.08 (0.23)
Surprise	**−0.16 (0.01)**	0.09 (0.18)	−0.08 (0.19)	0.08 (0.21)	0.02 (0.79)
Performance Index r(*p*)					
Anger	0.07 (0.29)	−0.10 (0.13)	0.03 (0.69)	−0.11 (0.09)	−0.12 (0.05)
Disgust	**0.24 (0.00)**	−0.06 (0.32)	0.05 (0.47)	−0.12 (0.08)	0.05 (0.45)
Fear	**0.16 (0.011)**	0.00 (0.97)	0.07 (0.28)	−0.12 (0.06)	0.01 (0.85)
Happiness	**0.18 (0.004)**	−0.07 (0.31)	0.04 (0.57)	−0.07 (0.28)	0.01 (0.88)
Sadness	0.08 (0.21)	0.01 (0.86)	0.11 (0.09)	−0.12 (0.06)	−0.03 (0.60)
Surprise	**0.15 (0.02)**	−0.07 (0.27)	**0.17 (0.009)**	−0.09 (0.15)	0.05 (0.39)

Significant outcomes in bold; *r* = Pearson’s correlation.

#### Accuracy

There was a significant positive partial correlation (adjusted for age) between *disgust* and both verbal fluency and the Verbal Working Memory Component. *Sadness* and *surprise* both showed positive partial correlations with the Verbal Memory Component. All these correlations were positive, suggesting that better performance in these cognitive areas is related to better accuracy for those specific emotions.

#### Reaction time

Reaction time to *fear* was not correlated with any of the non-emotional cognitive variables. Reaction time to *anger* showed a positive correlation with the Verbal Working Memory Component; that is, better verbal working memory performance was associated with slower reaction time to anger. Reaction time to *sadness* showed a positive partial correlation with the Spatial Working Memory Component, with better spatial working memory performance associated with increased reaction time to *sadness*. Reaction time to *disgust*, *happiness*, and *surprise* all showed negative partial correlations with verbal fluency, indicating that better verbal fluency was associated with faster reaction times for these emotions.

#### Performance index

*Anger* and *sadness* did not correlate significantly with any of the non-emotional cognitive variables. *Disgust*, *fear*, *happiness*, and *surprise* all showed significant positive correlations with verbal fluency, while *surprise* was significantly positively correlated with the Verbal Memory Component.

## Discussion

### Main findings

The analysis aimed to examine the association between age, mood disorder, and emotion processing. The main results were as follows:

1. Contrary to our hypothesis, there was no effect of mood disorder on FER. This was the case whether healthy people were compared with people with mood disorder (MDD and BD combined) or healthy people were compared with people who were currently depressed (all MDE).
2. As hypothesized, there was an interaction between age and emotion on all three outcomes (accuracy, reaction time, PI). Further examination of this showed that increased age was significantly associated with reduced accuracy and/or PI, and increased latency, in processing emotions of *anger, disgust, fear, sadness, and surprise*. In no analysis was age associated with an improvement in PI on these emotions. However, increased accuracy in recognizing *happiness* was seen with increased age, while there was no effect of age on reaction time or PI for *happiness*. As noted, these relationships were not modified by having a mood disorder or being currently depressed, which was contrary to our original hypotheses.
3. To explore the relationship between emotion processing and non-emotional cognitive function, the age-adjusted correlation between non-emotional cognitive function and FER performance was examined. The most consistent results were found for an association between verbal fluency and FER, with a positive relationship between verbal fluency and performance processing expressions of *happiness*, *fear*, *surprise,* and *disgust*, and a negative relationship between verbal fluency and speed of responding to expressions of *disgust*, *happiness,* and *surprise*.

### Influence of mood disorder diagnosis and mood state

Having a mood disorder diagnosis did not have a significant effect on ability to process emotions compared with people without such a diagnosis, nor did being currently depressed (MDE). For individual emotions, results for the three facets of FER were generally consistent, with processing of *happiness* either improving or not changing significantly with increased age, while processing of other emotions declined with age. In all cases apart from *anger*, mood disorder diagnosis did not modify the association between age and FER performance. It could therefore be concluded that there is a “positivity effect” (regardless of mood disorder diagnosis) whereby processing of emotions other than *happiness* declines, while processing of *happiness* remains stable or improves with age. An analysis of the comparison between currently depressed (MDE) participants and healthy control participants, showing no difference in FER between groups, has been reported previously, but without considering the effects of age (Bourke et al., 2012). Data in the BD group have not been reported previously. However, one previous study analyzing several FER tasks also found no overall performance difference from healthy controls in bipolar depression and euthymia (Robinson et al., 2015). The analysis of current depression (MDE) in the present study includes both the previously analyzed group and a further 37 people with bipolar depression. The MDD group was only ‘moderately’ depressed, with a mean MADRS score of 24, which may have mitigated against a significant group finding. It may also be the case that examining the misidentification of neutral stimuli is a more sensitive measure than emotion identification since previous research in mood disorders has shown the most consistent results in the misidentification of ambiguous faces as *sad* or attentional bias toward *sad* faces (Bourke et al., 2010). In the current analysis, misidentification was not examined, primarily because of the low number of neutral stimuli (only 24 neutral stimuli) that were presented in the paradigm used.

These results conflict with the theory that in mood disorders, the opposite of the positivity effect (i.e., a bias toward negative emotional stimuli) would be seen, which increases or maintains a vulnerability to lowered mood (Disner et al., 2011; Warren et al., 2015; Miskowiak et al., 2019). Such findings have been seen in both euthymic and symptomatic mood disorder populations, but have not been completely consistent, in part due to the range of paradigms used to measure emotion processing. However, Miskowiak et al. (2019) conducted a comprehensive literature review of emotion processing which found that specifically for FER, deficits were seen in 77% of studies of remitted patients with BD (17/22) and in 71% of studies in symptomatic patients with BD (10/14).

There are further caveats regarding data in the mood disorder samples from the current study. The BD group was relatively young, and as such conclusions cannot be drawn regarding the effects of advanced aging. In addition, the BD group was in a variety of mood states and emotion processing may be different among manic, depressive, and euthymic mood states. The number of participants in each state in the BD group was too small to undertake a sub-analysis, however, the influence of current depression (MDE) has been examined in the combined group. The finding of an interaction between mood disorder and age for *anger* is an outlier in these results (Supplementary Figure S1). Effect size differences between groups were small, suggesting that the results did not stem from a Type 2 error.

### Influence of age

For identification of emotions other than *happiness,* increasing age was generally associated with reduced accuracy and PI, and longer reaction time. Increased accuracy in identifying *happy* faces was associated with increasing age, and no significant change was found with age in reaction time or PI. The PI was designed to examine overall performance, accounting for both speed and accuracy. Thus, in these data, older people are more accurate at recognizing *happy* faces – but not quicker, and when these two variables are combined, “performance” is not improved. Our data suggest aspects of a positivity effect with increasing age. The results found are consistent with the findings of the Ruffman et al. (2008) meta-analysis for *fear*, where accuracy decreased with age. Regarding results related to *happy* faces, the Ruffman et al. meta-analysis did not find an increase in accuracy with increasing age–in fact, increasing age was associated with reduced accuracy, but less so for *happy* emotions. Of interest, the present study found a significant effect for *surprise* with age for accuracy, reaction time, and PI. Ruffman et al.’s meta-analysis did show a smaller worsening for *surprise*, alongside what they found for *happy*, and Hayes et al. (2020) showed that for reduced intensity photos only, *surprise* did show a reduction in accuracy with age. The image sets used in the two original studies in these analyses included images that varied in intensity. A major difference between the current study and Ruffman et al.’s study is the mean age of participants. Ruffman et al. (2008) examined much older participants with a mean age above 55 years, which is significantly older than the current sample–with a mean age of 25 years in the BD sample (maximum age 35) and in the MDD and healthy samples, a mean age of 38 years and maximum of 65 years.

Several previous studies have examined emotion recognition across the age range and have been undertaken in healthy populations. In contrast, the current study examined the effects of age in populations with mood disorders in comparison to a relatively small healthy control group. Therefore, these analyses cannot be seen as directly comparable to previous studies examining the effects of age on emotion processing. Such studies have tended to suggest a U-shaped curve for overall emotion recognition performance, with ability peaking in middle age and then declining (Horning et al., 2012; Olderbak et al., 2019). Olderbak et al. (2019) used an Identification of Emotion Expressions from Composite Faces Task and analyzed the performance overall, rather than by specific emotion. They found peak performance at 30 years of age with performance declining thereafter. Using a dynamic image task, Horning et al. (2012) found that for *fear*, *sadness*, and *happiness*, there was a peak in performance at 45 years of age and a decline in accuracy thereafter. In contrast, and more in keeping with our results and those of Ruffman et al.’s meta-analysis, West et al. (2012), also using a dynamic image task, found significant decline for *fear*, *anger*, and *sadness*, but no decline with age for *happiness*. In contrast to the current study, however, West et al. found no improvement in accuracy in recognizing expressions of *happiness* with age. West et al.’s study extended to a much greater age and the greatest declines were seen from the age of 60 years and onwards. Similarly, Kessels et al. (2014), using static images, found a linear decline with age that became significant in the 60s, particularly for *anger*, with relatively less decline for other emotions including *happiness*. Overall, the evidence is reasonably consistent that there is little decline in accuracy recognizing *happy* expressions compared with negative emotions, while in the current study, in a group consisting mainly of relatively younger (young adult/middle age) people with mood disorders, age was associated with an increase in accuracy of recognizing *happy* expressions. As noted, this is surprising given our hypothesis of a negative bias in people with a mood disorder.

### Relationship of emotional processing to non-emotional cognitive function

The Dynamic Integration Theory suggests that negative emotions are more effortful to process, and therefore, reduced cognitive functioning in older people results in a tendency to attend less to negative information (Labouvie-Vief, 2003). This suggests a relatively close relationship between emotional processing and non-emotional cognitive function. However, the current analyses found few outcomes of significance when examining the relationship between performance on FER (“hot” processing) and non-emotional cognitive function (“cold” processing). The emotion most clearly linked to cognitive functions was *disgust*; with accuracy in recognizing expressions of *disgust* correlating with performance on measures of verbal fluency (Controlled Oral Word Association Task) and Verbal Working Memory. Verbal fluency was the cognitive function most consistently linked to the processing of emotions, with a positive relationship with the PI, and negative relationships with reaction time, for *disgust*, *fear*, *happiness*, and *surprise*.

Generally, it is considered that crystalized knowledge such as word finding, procedural memory, or cultural knowledge is preserved in aging (Christensen, 2001). Verbal fluency, while requiring word finding, also involves executive functions to allow for specific letter- or category-based retrieval (Whiteside et al., 2016). A meta-analysis by Rodríguez-Aranda and Martinussen (2006) indicated that verbal fluency is negatively affected by age. Specifically, they found that this decline in verbal fluency changes most significantly after 60 years of age and continues thereafter. Associations found in the current analyses may suggest that some processes involved in emotion processing are linked to those used during verbal fluency tasks. However, it is important to note that verbal fluency is not particularly specific and includes components of cognition other than executive function, thus being particularly subject to the phenomenon of “task impurity” (Snyder et al., 2015).

Of note, in the current study, the age range of the sample was 16–65 years – so this does not provide evidence of a decline in cognitive processes that occur beyond this age, which may be accelerated. At a younger age, results are also likely to be affected by brain maturation. De Luca et al. (2003) examined executive function changes over the lifespan, suggesting that improvement in executive functioning capabilities correlates with increased myelination and diffuse synaptogenesis of frontal regions, which continues well into the second decade of life. The peak of performance of some executive tasks such as organization of goal-directed behavior is seen between 20 and 29 years (De Luca et al., 2003), meaning that maturation of this skill is likely to still be occurring for participants in the current study. Conversely, De Luca et al. (2003) also found that for those skills that mature in early adulthood, the decline is seen relatively early in the aging process, with some skills declining significantly in the 50-64-year-old age range.

### Limitations

As an examination of the effects of age on emotional processing in mood disorders and healthy people, the current study has several limitations. First, as mentioned in previous sections, the age range means that in some participants, brain maturation was likely still occurring. This may have resulted in effects that are quite different from those of “aging.” Second, the age range did not extend into more advanced aging beyond the age of 65. Third, there was a small number of neutral faces in the sample, meaning the misidentification of neutral faces was not able to be examined. This limitation is important, as misidentification shows the most consistent evidence of attentional biases in mood disorders (Bourke et al., 2010). When examining the hypothesis that declining executive function may impact on processing of negative emotions, the battery of cognitive tests was broad in nature, with relatively few tests in each domain. More detailed analysis using more cognitive tasks in each domain would be interesting, most especially in the verbal fluency, verbal memory, and verbal working memory (digit span) domains, all of which showed preliminary results of significance.

The analysis includes multiple tests of significance and there is therefore the risk of Type 1 errors. Rather than correct statistically for multiple testing we opted to examine the pattern of the results and to interpret significant results based on this. For example, in the analysis of the correlation between non-emotional cognition and FER, we have noted that the correlations between verbal fluency and aspects of FER are likely to be of importance, while the other statistically significant results are more likely to be chance findings.

### Strengths

The current study represents an initial exploration of the effects of age on emotional processing in healthy participants and in people with mood disorders aged 16 to 65 years. It is the first direct examination of the effects of age in a large (*n* = 198) sample of individuals with mood disorders. Of note is the use of a performance index to explore the composite of speed and accuracy involved in the performance of the FER task. We believe this is the first use of this in the facial emotion processing literature.

## Conclusion

The current study explored interactions between age and mood disorder on emotion processing, while also considering the effect of non-emotional cognitive function. A degree of positivity effect was demonstrated with increasing age, across an age range of 16–65 years, in healthy participants and participants with mood disorders (MDD and BD). There was no group difference in emotional processing regardless of whether mood disorder diagnosis or current depression (MDE) was examined. The positivity effect is seen here by late middle age, and in people with mood disorders, which is surprising given that findings in mood disorder samples have generally shown the opposite. A clear future direction would be to examine the relationship between mood disorder, age, and emotional processing in older-age samples (over 65 years).

## Data availability statement

The original contributions presented in the study are included in the article/Supplementary material, further inquiries can be directed to the corresponding authors.

## Ethics statement

The studies involving humans were approved by New Zealand Health and Disability Ethics Committee. The studies were conducted in accordance with the local legislation and institutional requirements. The participants provided their written informed consent to participate in this study.

## Author contributions

VG, WM, CF, and RP contributed to the conception and design of the study and performed the statistical analysis. VG and WM organized the database. VG and RP wrote the first draft of the manuscript. VG, WM, RP, and PG wrote sections of the manuscript. All authors contributed to manuscript revision, read, and approved the submitted version.
