# Supplementary material for: The effect of age on emotion processing in individuals with mood disorders and in healthy individuals
Source: Front Psychol. 2024 Jan 26;15:1204204. doi: 10.3389/fpsyg.2024.1204204 (PMC10853436; doi:10.3389/fpsyg.2024.1204204)

**Figure S1. Accuracy and Performance Index for A*nger* by Group (Healthy Control vs Mood disorder) and Age.**

**Figure S2. Scatterplots of Accuracy, Reaction Time, and Performance Index for Each Significant Interaction of Emotion by Age and Group from Table 2.**


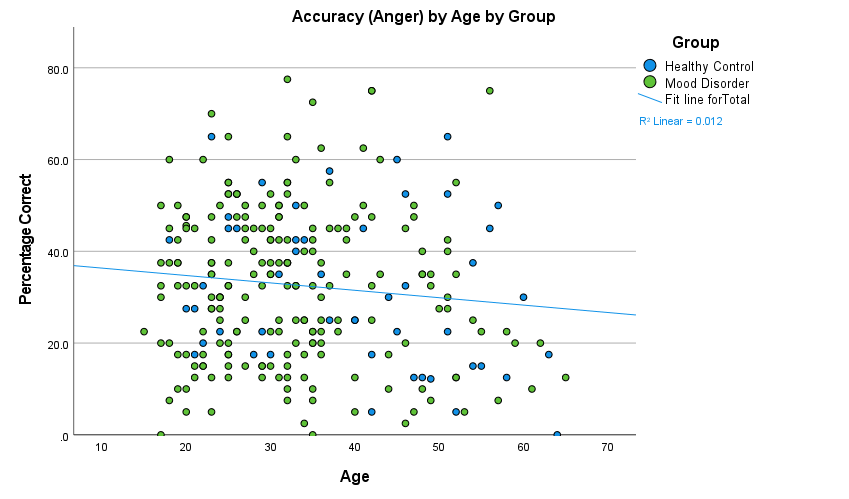


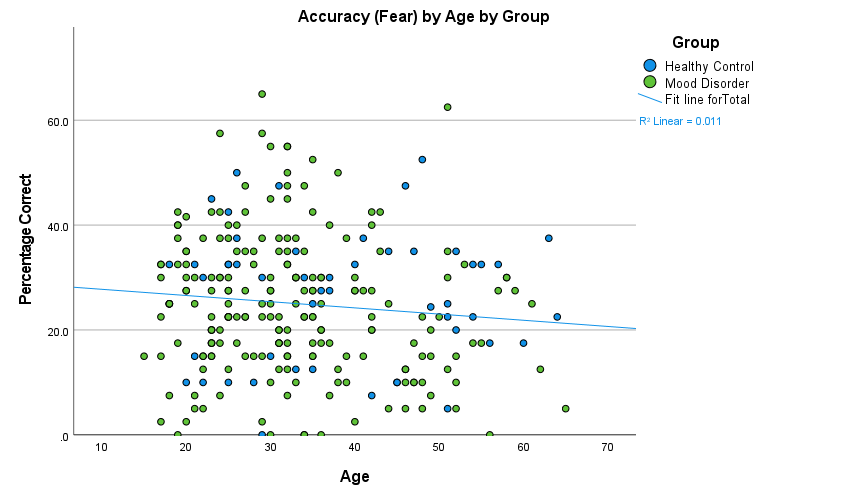


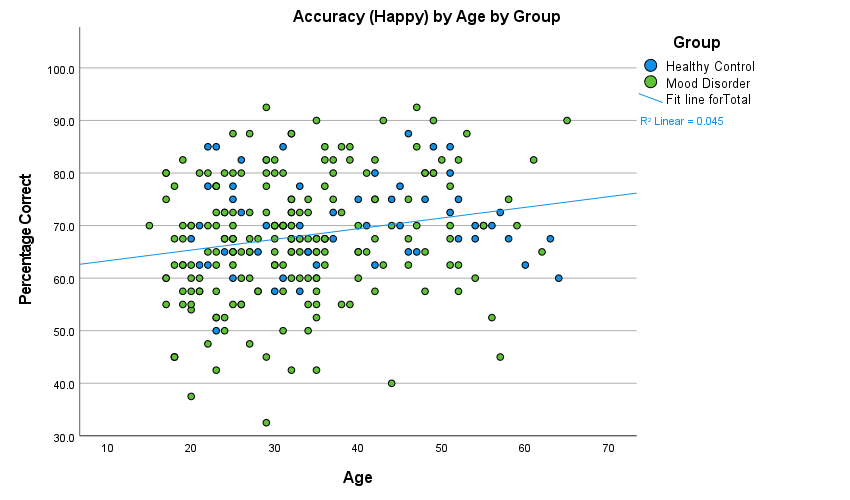


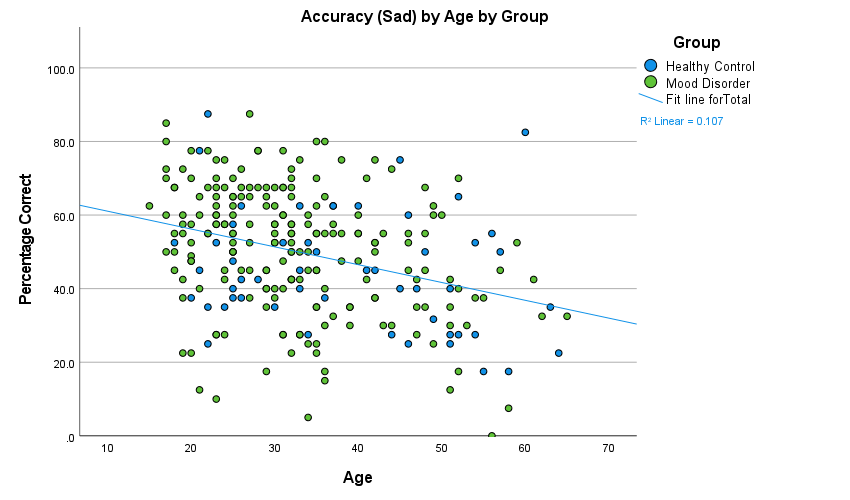


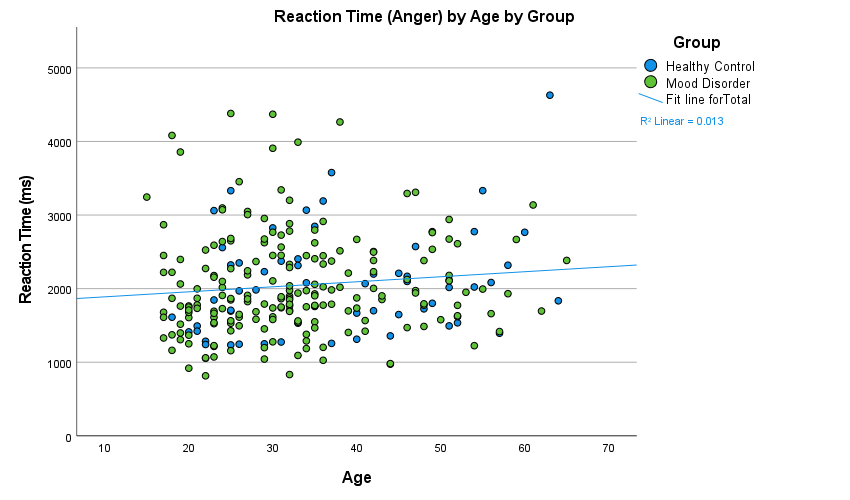


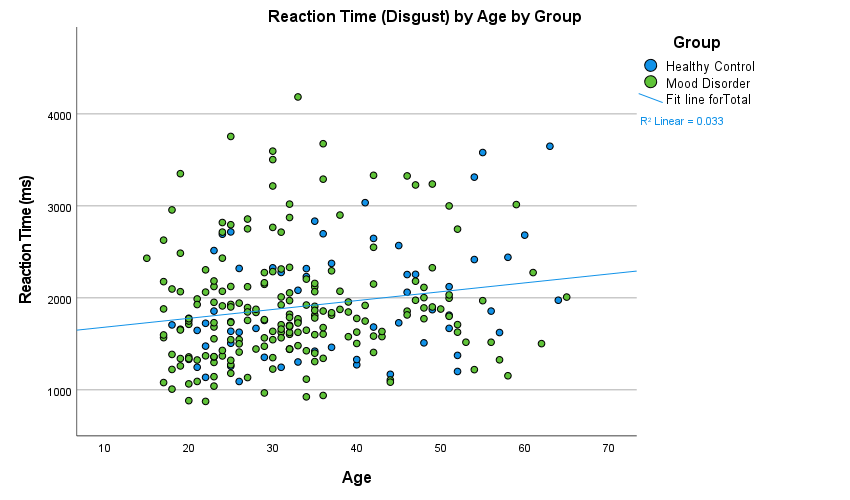


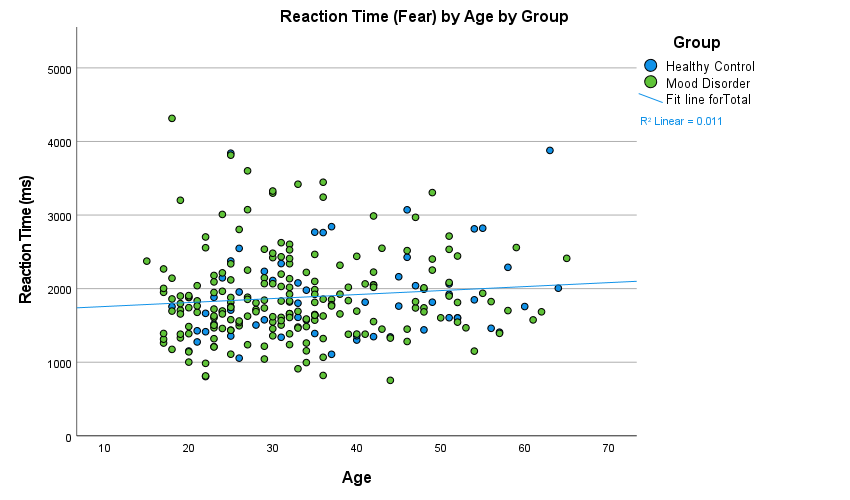


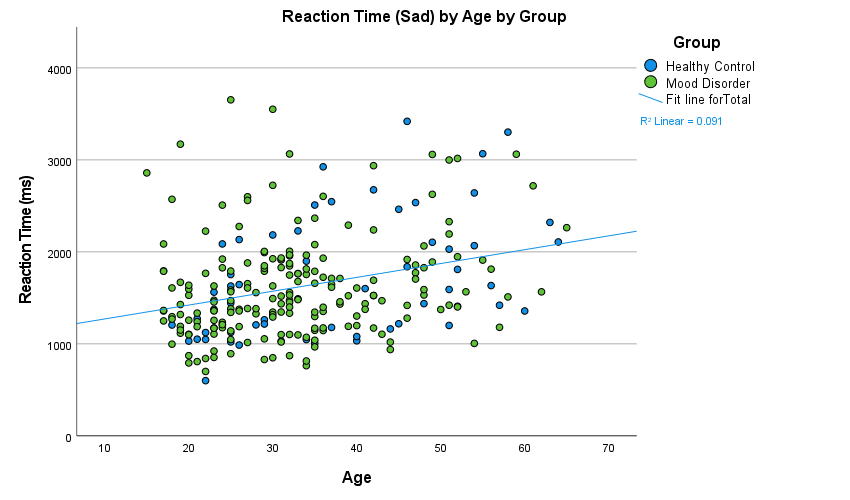


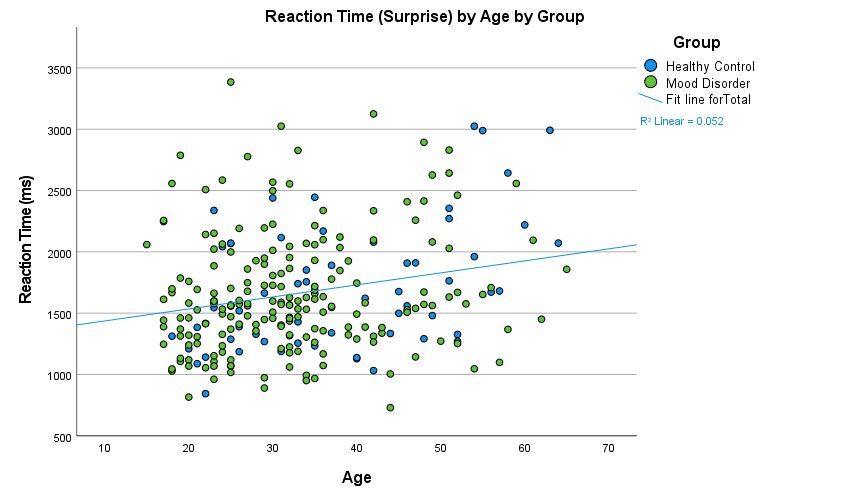


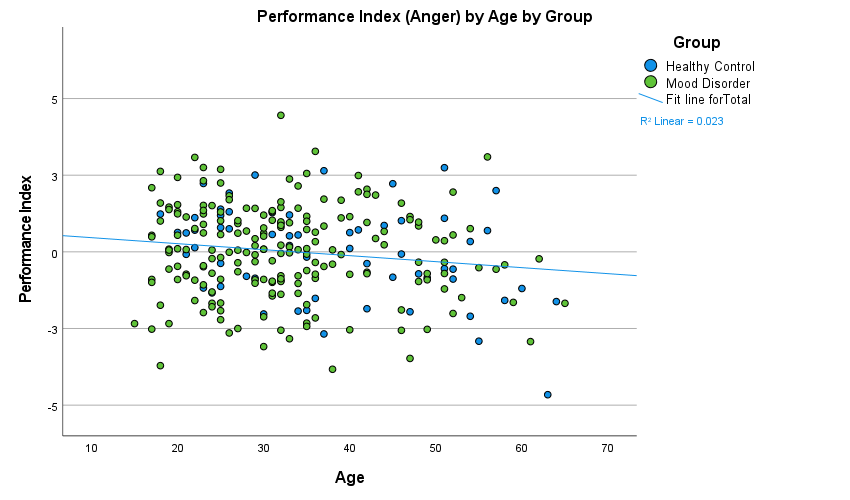


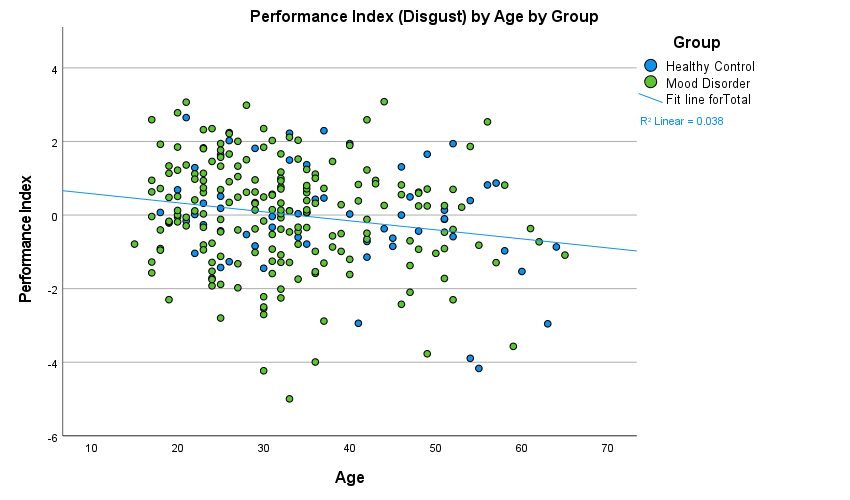


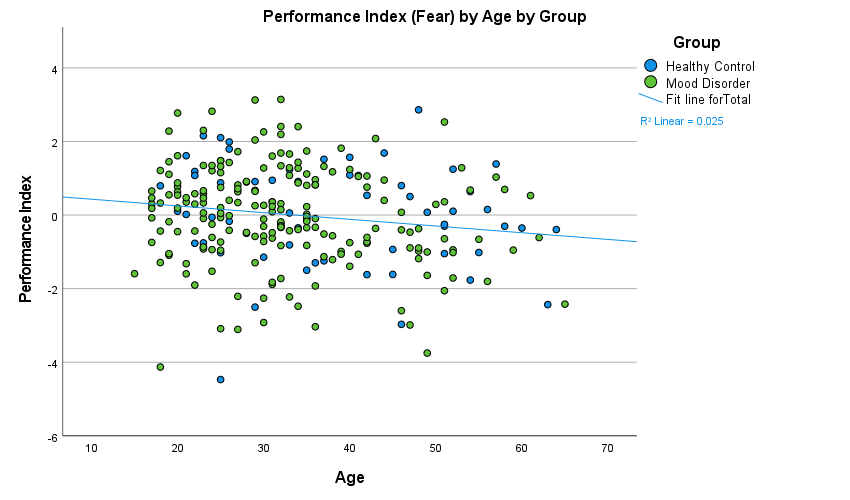


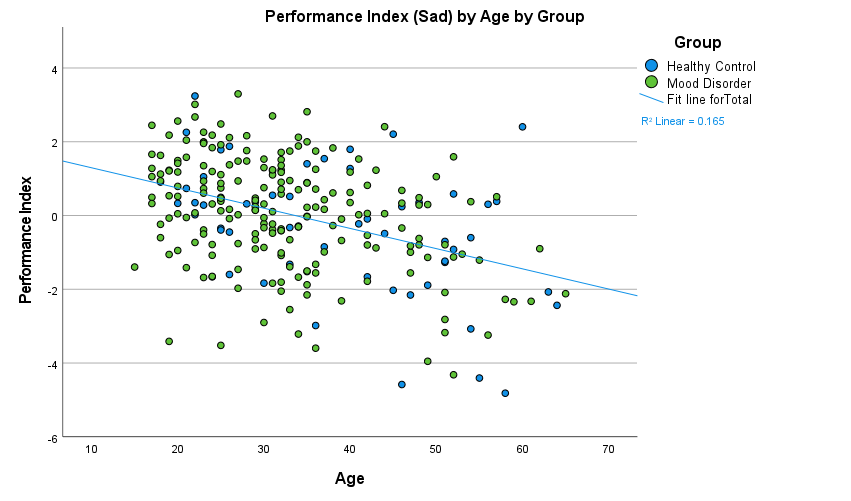


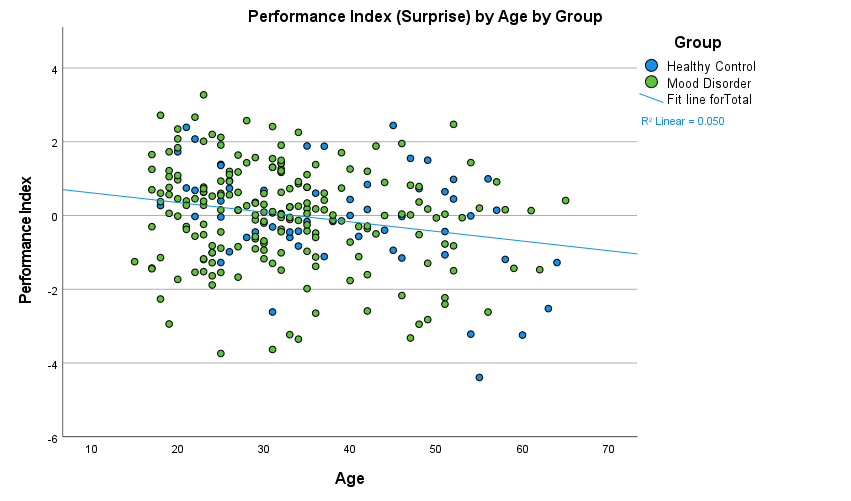

Supplement: Supplementary file 1 [file Data_Sheet_1.docx]
